# Single-Step Photochemical Formation of Near-Infrared-Absorbing Gold Nanomosaic within PNIPAm Microgels: Candidates for Photothermal Drug Delivery

**DOI:** 10.3390/nano10071251

**Published:** 2020-06-28

**Authors:** Sreekar B. Marpu, Brian Leon Kamras, Nooshin MirzaNasiri, Oussama Elbjeirami, Denise Perry Simmons, Zhibing Hu, Mohammad A. Omary

**Affiliations:** 1 Department of Chemistry, University of North Texas, Denton, TX 76203, USA; briankamras@my.unt.edu (B.L.K.); nooshinmirzanasiri@my.unt.edu (N.M.); elbjeirami@unt.edu (O.E.); 2 Department of Mechanical and Energy Engineering, University of North Texas, Denton, TX 76203, USA; denise.simmons@unthsc.edu; 3 Department of Physics, University of North Texas, Denton, TX 76203, USA; Zhibing.Hu@unt.edu

**Keywords:** nanoparticles, anisotropy, plasmonic photothermal therapy, surface plasmon resonance, light scattering, cancer treatment, drug release, microgels

## Abstract

This work demonstrates the dynamic potential for tailoring the surface plasmon resonance (SPR), size, and shapes of gold nanoparticles (AuNPs) starting from an Au(I) precursor, chloro(dimethyl sulfide)gold (I) (Au(Me_2_S)Cl), in lieu of the conventional Au(III) precursor hydrogen tetrachloroaurate (III) hydrate (HAuCl_4_). Our approach presents a one-step method that permits regulation of an Au(I) precursor to form either visible-absorbing gold nanospheres or near-infrared-window (NIRW)-absorbing anisotropic AuNPs. A collection of shapes is obtained for the NIR-absorbing AuNPs herein, giving rise to spontaneously formed nanomosaic (NIR-absorbing anisotropic gold nanomosaic, NIRAuNM) without a dominant geometry for the tesserae elements that comprise the mosaic. Nonetheless, NIRAuNM exhibited high stability; one test sample remains stable with the same SPR absorption profile 7 *years post-synthesis thus far*. These NIRAuNM are generated within thermoresponsive poly(*N*-isopropylacrylamide) (PNIPAm) microgels, without the addition of any growth-assisting surfactants or reducing agents. Our directed-selection methodology is based on the photochemical reduction of a light-, heat-, and water-sensitive Au(I) precursor via a disproportionation mechanism. The NIRAuNM stabilized within the thermoresponsive microgels demonstrates a light-activated size decrease of the microgels. On irradiation with a NIR lamp source, the percent decrease in the size of the microgels loaded with NIRAuNM is at least five times greater compared to the control microgels. The concept of photothermal shrinkage of hybrid microgels is further demonstrated by the release of a model luminescent dye, as a drug release model. The absorbance and emission of the model dye released from the hybrid microgels are over an order of magnitude higher compared to the absorbance and emission of the dye released from the unloaded-control microgels.

## 1. Introduction

Stimuli-responsive polymers, hydrogels, and microgels are appealing to biomedical researchers as potential drug delivery candidates, based upon their ability to undergo phase transitions in response to various environmental factors (e.g., temperature, light, pH, and/or ionic strength). High water content, hydrophilicity, softness, flexibility, biocompatibility, and properties similar to biological tissues render such polymer hydrogel materials as suitable platforms for various biomedical applications [1,2,3,4,5,6,7]. To extend their functionality, numerous investigations have demonstrated the loading and/or stabilization of different nanomaterials such as metallic nanoparticles and quantum dots within these microgels [8]. Poly(*N*-isopropylacrylamide) (PNIPAm) is often used because of its thermoresponsiveness results in “smart gel” materials. PNIPAm, which has a lower critical solution temperature (LCST)–32 °C, can be tuned by varying its composition and crosslinking density [9]. In a related area, AuNPs are frequently studied for their structural, optical, electronic, and catalytic properties. Among those, AuNPs with anisotropic shapes (e.g., rods, triangles, cubes, truncated octahedra, shells, stars, etc.) with surface plasmon resonances (SPR) that extend beyond the visible region are desirable for sensing and plasmonic photothermal therapy (PPTT) applications [10,11,12].

### 1.1. Overview of Anisotropic AuNPs Usage

By regulating the size and shape of a given anisotropic gold nanoparticle, the NIR SPR resonance band can be tuned to lie within the “near-infrared window” (NIRW) that lies within 700–1300 nm [13]. Due to their strong NIR absorption and high extinction coefficient [12,14,15], anisotropic AuNPs are highly appealing candidates for plasmonic photothermal therapy (PPTT) [15]. Usually, researchers exploiting the plasmonic photothermal properties or localized sensitive properties of Near infrared gold nanoparticles (NIRAuNPs) use gold nanorods (AuNRs) stabilized in the highly cytotoxic (vide infra) surfactant reagent cetyltrimethylammonium bromide (CTAB) made using the classic seed-mediated method, developed by the Murphy group and later modified by El-Sayed et al. [16]. In addition to the classic seed-mediated method, there are also seedless and polyol processes developed by numerous researchers [17]. For example, Huang et al. produced gold nanorods (AuNRs) with high aspect ratios by employing silver bromide (AgBr) as a capping agent, CTAB as the growth-directing surfactant, and HCl to modulate pH [17]. Wang et al. used 5-Bromosalicylic acid as a templating factor in addition to the typical CTAB surfactant and silver nitrate as a capping agent, which produced rods at a practically quantitative yield [18]. In addition to AuNRs, other anisotropic shapes including nanoplates, nanotriangles, nanocubes, and nanostars with sharp edges and NIR SPR features are equally attractive candidates for biosensing and surface-enhanced Raman scattering (SERS) applications [19]. These anisotropic particles are mainly synthesized using protocols resembling the AuNR synthesis [6,20].

### 1.2. Toxicity Challenges

The abovementioned methods involve surfactants such as CTAB or silver ions, which are both reported to be toxic by various research groups [21]. Even without CTAB, overcoming the inherent toxicity of silver ions is still challenging [21]. The toxicity of CTAB and the role of CTAB in AuNRs synthesis have been detailed by many groups [22,23]. In 2017, Bandyopadhyay illustrated that the positive surface charge of CTAB induces cytotoxicity and hinders the adsorption of serum proteins onto the particle surface, which can alter the process of endocytosis [24]. The role of CTAB in complicating the biomedical applications of AuNRs was explained by Kohanloo et al., who determined the critical CTAB:nanorod concentration ratio to be approximately 740,000:1 to attain nanorods stability upon the formation of a CTAB double-layer, whereas experimentally, an even larger excess of CTAB is added [25]. Additionally, Choi et al. demonstrated the necessity of replacing or removing CTAB for in vivo application of AuNRs. Currently, CTAB toxicity is circumvented by ligand exchange using thiolated polyethylene glycol (PEG-SH) because of the latter’s water solubility, biocompatibility, and relatively higher binding affinity for Au than CTAB [26]. Although gold nanoparticle cores alone are shown to be nontoxic in numerous in vitro studies, the stabilizing ligand can be toxic. Therefore, differentiating the toxicity due to the stabilizing ligands versus the gold core itself is critical. These potential problems and risks are high with anisotropic AuNPs due to their highly exposed surfaces and defects compared to spherical AuNPs [21].

### 1.3. Nontoxic Tuning Methods

Among the few methodologies that do not use surfactants or CTAB, Guo and coworkers have shown success using a seed-mediated growth approach to create size-tunable AuNPs in the range of 5 to 200 nm by reduction of Au(III) with sodium borohydride (NaBH_4_) and hydroxylamine hydrochloride (NH_2_OH·HCl) [27]. Alternatively, some research groups have explored “green” synthesis methods to overcome cytotoxicity issues and the tedious ligand exchange processes inherent to conventional syntheses of various NIRAuNPs [26]. Biological extracts are known to be employed as reducing/stabilizing substances for making both spherical and anisotropic AuNPs. Among them, the biological synthesis of triangular gold nanoprisms, stabilized in lemongrass by Sastry et al., has attracted immense interest [28]. Sakthivel and coworkers have used *Coleus amboinicus* extracts as reducing and stabilizing agents for producing different sizes of spherical, polydispersed AuNPs [29]. Kitching et al. described the advantages of fungal biosynthesis of gold nanoparticles and have also explained the complications involved in understanding the biosynthetic mechanism of AuNPs formation within biological media [30]. In 2014, the Menon group successfully synthesized NIR-absorbing anisotropic AuNPs using a cocoa extract and explained the challenges involved in making AuNPs with NIRW-absorbing features within these biological [31].

### 1.4. Photochemical Formation of AuNPs

Numerous literature examples have demonstrated the photochemical formation of silver nanoparticles, but an extension of this methodology to AuNPs is still limited. This is likely due to easy access to the light-sensitive silver precursors, which contrast with the relative stability of the hydrogen tetrachloroaurate (III) hydrate (HAuCl_4_) precursor. However, there are a few examples of spherical and anisotropic AuNPs made from HAuCl_4_ precursors by employing photochemistry. The Zhao group demonstrated the formation of small, spherical AuNPs by irradiation of HAuCl_4_ with a 300 nm wavelength light source [32]. In 2005, the El-Sayed group demonstrated the formation of Au(0) with continuous excitation of HAuCl_4_ in the presence of ethylene glycol and polyvinylpyrrolidone (PVP) using a 250–400 nm light source [33]. Pal has demonstrated the photochemical formation of spherical AuNPs in the presence of sodium dodecyl sulfate (SDS) and dopamine hydrochloride [34]. There are even fewer literature examples illustrating the photochemical formation of anisotropic AuNPs. Among them, the photochemical synthesis of gold nanorods by Kim et al. is notable for tuning the aspect ratio of AuNRs by using photochemistry in the presence of CTAB and silver ions [35]. Zhu et al. have shown the photochemical formation of a mixture of anisotropic AuNPs within room-temperature ionic liquids (RTILs) [36]. In 2016, Wei et al. demonstrated a plasmon-driven synthesis of Au nanoprisms within PVP [37]. Analyzing the growth of AuNRs by photochemical means, some researchers have also analyzed the role of CTAB, silver ions, and photoinitiators [38]. Pignatelli et al. found that varying the irradiation time, wavelength, and intensity can tune the absorption and aspect ratio of AuNRs [39].

### 1.5. Stabilization of AuNPs within PNIPAm Hydrogels/Microgels

Several groups have modified AuNPs, postsynthesis, by binding PNIPAm or surface-functionalized PNIPAm to the AuNPs surface and found that the resulting hybrid material exhibits a thermoreversible phase shift and corresponding color-absorption shift [40]. Despite the synergistic properties of temperature sensitivity and heat-generating ability, only a handful of demonstrations exist that incorporate AuNRs or NIR-absorbing AuNPs within PNIPAm microgels or hydrogels. Zhao et al. loaded CTAB-stabilized AuNRs into a triblock copolymer of randomly arranged acrylamide, acrylonitrile, and dimethylacrylamide to exploit the polymers’ upper critical solution temperature (UCST); such methods afforded a temperature-responsive, “switchable” composite material [41]. This composite, when irradiated with visible light, resulted in the release of an adsorbed protein from within the pores of the composite. Liz-Marzan et al. found that CTAB-stabilized AuNRs adsorbed within PNIPAm microgel spheres undergo a phase-transition, which red-shifts the rod’s SPR when irradiated with NIR light [42]. Kawano et al. have demonstrated photothermal heating using CTAB stabilized, silica-coated AuNRs within PNIPAm nanogels [43]. In 2010, Jiang et al. demonstrated the formation of PNIPAm-AuNR composites by using the seed-mediated growth method; they reasoned that such PNIPAm composites have great advantages in surface-enhanced Raman scattering (SERS) as these composites combine the thermoresponsive behavior of microgels and the SERS effect of AuNRs [44]. In 2015, F.-Lopez et al. have demonstrated reversible plasmon coupling from AuNRs with differing aspect ratios doped within PNIPAm [45]. However, most or all of these methods, to the best of our knowledge, are based on loading or tethering premade anisotropic AuNPs or AuNRs to PNIPAm polymers or hydrogels [40,41,42,43,44,45].

### 1.6. Formation of AuNPs from Au(I) Precursor

Our literature search found a handful of studies investigating Au(I) species as AuNP precursors. In 1988, Vogler et al. formed AuNPs by reducing gold azide complexes via photolysis [46]. In 2004, Lee et al. demonstrated the formation of spherical AuNPs from Au(I)-SC18–alkane thiolates by electron beam irradiation [47]. Another important demonstration of using Au(I) compounds for the synthesis of gold nanostructures comes from the 2008 report by the Xia group, using Au(I) halides including AuCl and AuBr to make AuNPs in chloroform in the presence of alkylamines with heating at 60 °C. The method demonstrated that the relatively low stability of Au(I) halides resulted in AuNP formation in the absence of any reducing agents [48]. Included in this handful of reports, our group demonstrated for the first time, the aurophilicity and ligand π-acceptance ability to sensitize the photoreactivity of Au(I) complexes. In 2007, we reported our findings on the propensity of Au(I) isonitriles to form AuNPs by photolysis in the presence of polyamidoamine (PAMAM) dendrimers in organic media [49]. These findings led to the discovery of Au(I) isonitriles transition through an Au(0) intermediate and present a possible mechanism for the formation of AuNPs. We continue to build upon our findings, and to the best of our knowledge, we report herein, the very first photochemical synthesis method for selectively making strong NIRW-absorbing AuNPs comprising a mixture of anisotropic shapes. Our method uses an Au(I) precursor within PNIPAm-*co*-allylamine microgels medium in the complete absence of any synergetic reducing agents or surfactants. To evaluate the wide range potential of this methodology, our work involves the formation of different sizes of spherical AuNPs, ranging from 5 to 50 nm, within different types of stabilizing media, again in the complete absence of any additional (potentially toxic) chemicals. The stabilizing media types included: agarose, alginic acid, chitosan, hydroxypropyl cellulose (HPC), polyvinyl alcohol (PVA), polyacrylic acid (PAA), PNIPAm microgels, and sodium dodecyl sulfate (SDS).

## 2. Materials and Methods

*N*-isopropylacrylamide (NIPA) monomer, allylamine, acrylic acid, *N,N’*-Methylenebis(acrylamide) (BIS), potassium persulfate (KPS), and sodium dodecyl sulfate (SDS) were purchased from Polysciences Inc., Warrington, PA, USA). Ethanol (99.5%), diethyl ether, dimethyl sulfide (Me_2_S), 85% deacetylated medium-molecular-weight chitosan, 50,000 Mw 30% polyacrylic acid, 100,000 Mw alginic acid sodium salt, 80,000 Mw hydroxypropyl cellulose, 90,000 Mw poly(vinyl alcohol), 96% bovine serum albumin (BSA), and wide-range agarose were bought from Sigma-Aldrich (St. Louis, MO, USA). Dialysis tubing (10 k MWCO) was purchased from California Biological (Burlington, NC, USA). All chemicals are used as received. For all experiments, 18.2 MΩ-cm (at 25 °C ) ultrapure water ( MilliporeSigma, Burlington, MA, USA)) was used. All glassware were cleaned in a bath of freshly prepared aqua regia, and were then rinsed thoroughly with ultrapure water. There were multiple samples made throughout the years using the same synthesis protocol and likewise for the experimental measurements below–all of which have attained high reproducibility.

### 2.1. Physical Measurements

A 450-W medium-pressure immersion mercury vapor lamp (Ace Glass) was placed into a photochemistry chamber equipped with a magnetic stirrer and a variable-temperature hot plate was used for all photochemical reactions. Based upon the manufacturer’s specifications of the total output energy from the mercury lamp, approximately 40–48% is in the ultraviolet region, 40–43% is in the visible region, and the remainder is in the infrared region. Regular borosilicate (BS) and quartz (SQ) silica glass were selectively utilized for the different syntheses. The same lamp source setup was employed for the synthesis of both spherical and anisotropic gold nanoparticle compositions. Absorption spectra were acquired using a Perkin-Elmer Lambda 900 double-beam UV/VIS-NIR spectrophotometer. The size, morphology, dispersity, and elemental analysis of different gold nanoparticle samples were determined using a high-resolution analytical transmission electron microscope (TEM). Specifically, a FEI Co. Tecnai G2 F20 S-TWIN 200 keV field-emission scanning transmission electron microscope (STEM) was used. A 1-nm STEM probe allowed for an imaging resolution of 0.19 nm, and a high angle annular dark field detector (HAADF) allowed for Z-contrast imaging in STEM mode at high resolution. High-resolution analytical capabilities were provided on the F20 STEM, which was equipped with an energy-dispersive X-ray spectrometer (EDS, also known as EDX), and a Gatan Tridiem parallel electron energy loss spectrometer (EELS) with a 2k × 2k CCD for energy-filtered imaging and high-rate spectrum imaging EELS. Unless otherwise mentioned, all images were collected in a bright field mode. Scanning electron microscopy (SEM) images were collected using an FEI Nova 200 NanoLab, which is a dual column ultrahigh resolution field emission scanning electron microscope. The nanoscale chemical analysis was performed with an EDS system with spectrum imaging control.

### 2.2. Syntheses of Gold(I) Precursor

The chloro(dimethyl sulfide)gold(I) complex (Au(Me_2_S)Cl) was synthesized by slightly modifying our earlier-established procedure described elsewhere [50]. Tetrahydrothiophene (THT) was replaced with dimethyl sulfide (Me_2_S) in the procedure. Before use, the glassware was oven-dried overnight at 150 °C. All manipulations were carried out under an atmosphere of purified nitrogen using the standard Schlenk technique. Briefly, the procedure involved 2 steps. In the first step, 1.8 g of gold (from the gold coin) was dissolved in 12 mL of aqua regia (9 mL of HCl and 3 mL of HNO_3_) in a 100 mL Schlenk flask. The reaction mixture was heated at 70 °C with continuous stirring to dissolve the solid gold. After dissolving the gold, heating was continued for an additional 30 min, followed by the addition of 3 mL of HCl. At this stage, heating was discontinued, and 30 mL of ethanol and 20 mL of water were added. The reaction was allowed to reach ambient temperature. At this stage, a yellow-orange colored solution was obtained. In the second step, the drop-wise addition of 2 mL of Me_2_S ligand resulted in the formation of a white precipitate. The reaction flask was then cooled in an ice bath, and the precipitate (product) was collected by sequential filtration with cold ethanol, followed by cold ether. The product was then dried overnight under vacuum. It should be noted that this heat-, light-, and water-sensitive Au(I) complex is always stored refrigerated and in the dark (foil wrap) away from the refrigerator’s light source.

### 2.3. Syntheses of PNIPAm Microgels

The hydrogel nanoparticles were made using the free radical precipitation method [50,51,52,53,54]. Precisely, 3.814 g of N-isopropylacrylamide monomer, 0.2 g of either allylamine or acrylic acid (for PNIPAm-*co*-allylamine and PNIPAm-*co*-acrylic acid, respectively), 0.066 g of BIS, and 0.08 g of SDS were dissolved in 245 g of ultrapure water. The solution was stirred under an N_2_ atmosphere for 40 min at 60 °C, and then, 5.0 mL of KPS (0.166 g) was added to initiate radical polymerization. Heating was continued for 5 h under a nitrogen atmosphere at 60 °C. The resulting PNIPAm-*co*-allylamine and PNIPAm-*co*-acrylic acid colloidal particles were transferred to 10,000 MWCO dialysis tubing and dialyzed against deionized (DI) water for 1 week at ambient temperature, followed by further purification via ultracentrifugation. The hydrodynamic radius (R_h_) of the microgels with and without AuNPs is measured by the dynamic light-scattering (DLS) technique. A commercial laser light-scattering (LLS) spectrometer (ALV/DLS/SLS-5000, Langen, Germany) equipped with an ALV-5000 Digital Time Correlator was used with a He–Ne laser (Uniphase 1145P, output power = 22 mW, and *λ=* 632.8 nm) as the light source. All size measurements were performed at a scattering angle of 90°. The volume shrinkage studies were conducted by controlling the temperature of the samples by a circulating water bath (Brinkmann Lauda Super RM-6, American Laboratory Trading, East Lyme, CT, USA) to within 0.02 °C. The samples for all the dynamic light scattering analysis were prepared by homogenization followed by dilution with Millipore water. Each sample was measured three times, and the mean radius was reported. The zeta potential was measured on a Zetasizer Nano ZS (Malvern Instruments, Westborough MA, USA) by loading samples into maintenance-free cells. Figure S1 shows the initial size of the PNIPAm microgels with and without AuNPs.

### 2.4. Syntheses of Spherical AuNPs within PNIPAm Microgels

In a typical procedure, a particular weight percent concentration of PNIPAm solution (2.0% w/v) is initially diluted to 0.2% w/v with ultrapure water and homogenized by stirring at ambient temperature (22 °C). Au(Me_2_S)Cl (5.0 mg, 1.68 µmol) is directly added to the PNIPAm microgel solution and stirred at ambient temperature for 5–10 min until all the Au(I) powder is homogeneously dispersed in the microgel medium. The freshly prepared PNIPAm-Au dispersion is immediately transferred into an SQ glass vial followed by irradiation in the photochemistry chamber equipped with a medium-pressure immersion mercury lamp as described in methods. All PNIPAm microgel samples containing the Au(I) complex are irradiated at ambient temperature. The temperature of the photochemistry chamber is maintained at 22 °C during the entire course of irradiation. For testing the effect of ambient conditions (room light and ambient temperature), the initial microgel solution containing the Au(I) precursor was transferred into a BS glass vial and then subjected to stirring at ambient temperature and ambient light. In all cases, AuNPs formation within the PNIPAm microgels was monitored by sampling the reaction solution over time and observing the recorded changes in absorption spectra. Depending upon the reaction conditions and the nature of the PNIPAm microgels (co-allylamine or co-acrylic acid), the total reaction time varied from 30 to 180 min. The absorption spectra were collected after placing the samples into 1-cm path length quartz cuvettes. For calculating the photochemical quantum yield of AuNPs within PNIPAm microgels, the changes in absorption of the samples with respect to time were analyzed.

### 2.5. Synthesis of Spherical AuNPs within Different Media

In a typical procedure, 1.0% *w/v* of a stabilizing media (alginic acid, chitosan, poly(acrylic acid), poly (vinyl alcohol), hydroxypropyl cellulose, sodium dodecyl sulfate, and bovine serum albumin) is dissolved in ultrapure water and homogenized for 10 min. Au(Me_2_S)Cl (5.0 mg, 1.68 µmol), is added to each of the abovementioned media, and the solution is subjected to photoirradiation in an SQ or BS glass container or heated at 37 °C. The reaction time varied for each solution depending upon the stabilizer used. Each reaction was stopped when the absorbance of AuNPs on the UV/VIS–NIR spectrophotometer ranged from 1.0 to 1.5 units (an arbitrary but consistent scale).

### 2.6. Synthesis of Anisotropic AuNPs (NIRAuNM) within PNIPAm-Co-allylamine Microgels

In a typical procedure, a particular weight percent concentration of the PNIPAm-*co*-allylamine solution (2.0% *w/v*) was initially diluted to 0.5% w/v with ultrapure water and homogenized by stirring at ambient temperature. The pH of the diluted microgel solution was adjusted to 4.0 by the dropwise addition of 0.1 M acetic acid (0.5 mL). Au(Me_2_S)Cl (3.0–9.0 mg, 1.02–3.02 µmol) was then added to the PNIPAm microgel solution and stirred at room temperature for 5–10 min until all the Au(I) powder is homogeneously dispersed in the microgels medium. The freshly prepared PNIPAm-Au solution was immediately transferred into a (BS) glass container followed by irradiation in the photochemistry chamber equipped with a medium-pressure immersion mercury lamp. For photoirradiated samples, the temperature was maintained between 0 and 5 °C throughout the reaction. For samples prepared by thermolysis, the PNIPAm microgel solution containing Au(Me_2_S)Cl was initially heated in a water bath at 37 °C for 10 to 15 min. After the initial color change, the reaction beaker was immediately transferred into an ice-water bath, and the temperature maintained between 0 and 5 °C throughout the remainder of the reaction time. NIRAuNM formation within the PNIPAm-*co*-allylamine microgel media was monitored by observing the recorded changes in absorption spectra as a function of time. Depending on reaction conditions, the total reaction time varied from 45 to 90 min. The absorption spectra were collected by collecting the samples into 1-cm path length quartz cuvettes. In all cases, the size, shape, distribution, and morphology of the AuNPs were determined using a field-emission scanning electron microscopy (FE-SEM, an FEI Co, Hillsboro, OR, USA.) and transmission electron microscopy (TEM, an EFI Co, Hillsboro, OR, USA). The samples were prepared by the drop-cast method on respective grids. The elemental composition of AuNPs was determined from EDS measurements.

### 2.7. Preparation of Samples for Electron Microscopy

For SEM, the microgel samples loaded with AuNPs were directly deposited on to SEM stubs and dried overnight inside a vacuum chamber. The samples were diluted with DI water as required for better microscopy results. For TEM, diluted hybrid microgel samples were added drop-wise on to the formvar carbon support copper grids and dried in a vacuum chamber for an hour. All samples for microscopy were stored strictly inside closed boxes to prevent any contamination. The SEM and TEM grids were obtained from Ted Pella, Redding, CA, USA.

## 3. Results and Discussion

### 3.1. Potential of Au(I) Precursor as an Alternative Precursor to Au(III) Salts for Making AuNPs

**Figure 1:**
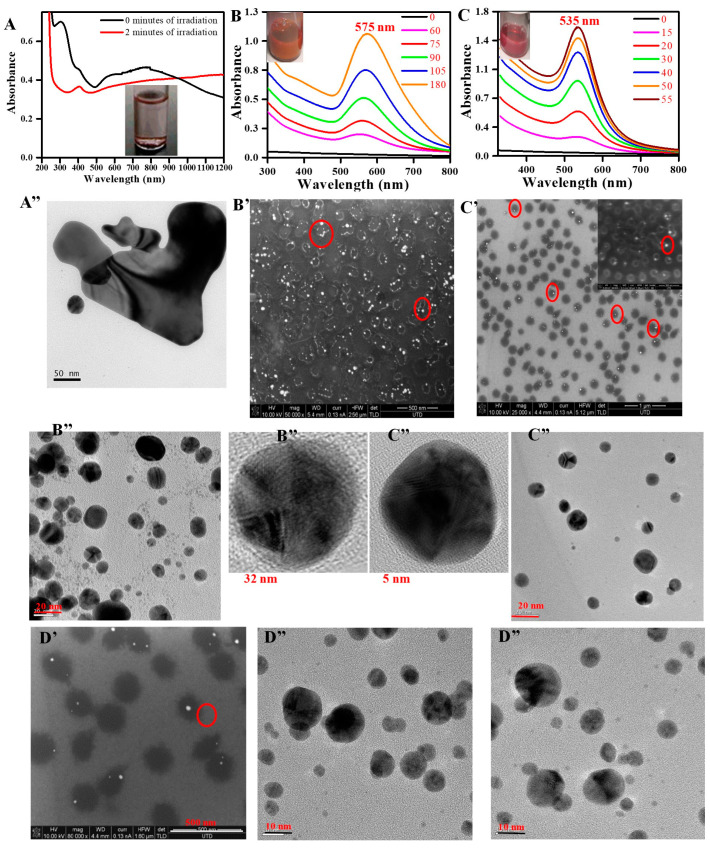
Absorption spectra and electron microscopy images of spherical gold nanoparticles (AuNPs) formed using chloro(dimethyl sulfide)gold (I) (Au(Me_2_S)Cl) precursor under different irradiation conditions within deionized (DI) water and poly(*N*-isopropylacrylamide) (PNIPAm) microgels. Traces (**A**,**A’’**) illustrate absorption spectra and TEM images of decomposed Au(I) in DI water. Traces (**B**,**B’**,**B’’**) depict absorption changes vs. time (minutes), SEM and TEM images for AuNPs formed under ambient light within PNIPAm-*co*-allylamine microgels. Traces (**C**,**C’**,**C’’**) represent absorption changes vs. time (minutes), SEM, and TEM images for AuNPs formed by photoirradiation within PNIPAm-*co*-allylamine microgels. Traces (**D’**,**D’’**) show SEM and TEM images of AuNPs formed by photoirradiation within PNIPAm-*co*-acrylic acid microgels. Refer to Figure S3 for the corresponding absorption spectra of sample (**D’**,**D’’**).

**Scheme 1:**
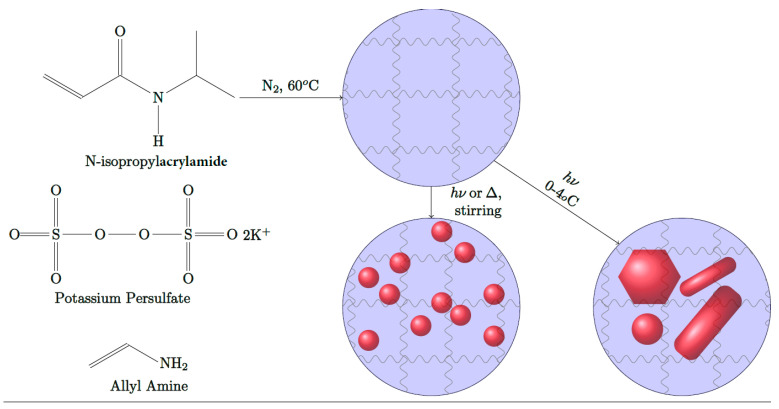
Synthesis of poly(*N*-isopropylacrylamide) (PNIPAm)-co-allylamine microgels, followed by the formation of spherical and anisotropic gold nanoparticles (AuNPs) within the microgels (hυ and Δ represent light and heat, respectively). The heating is performed at 37 °C for 10–15 min. The irradiation times are included in the actual data figures.

The schematics representing the in situ formation of both visible absorbing spherical AuNPs and NIR-absorbing anisotropic AuNPs within PNIPAm microgels are shown in Scheme 1. The one-electron photoreduction of Au(I) to Au(0) proceeds rapidly and spontaneously at ambient temperature in aqueous media. Compared to conventional Au(III) systems, the photochemical reduction of Au(Me_2_S)Cl in water was so spontaneous that it resulted in aggregated or decomposed Au(0) within a few minutes of irradiation (Figure 1A)– indicating its sensitivity to light. The broad SPR peak spreads across 550–900 nm, the faint red color of the Au(I)-water solution at 0 min irradiation, and the aggregation of Au(0) as revealed from the changes in absorption spectra (Figure 1A) within 2 min of irradiation all indicated both water and light sensitivity of Au(Me_2_S)Cl. After 2 min of irradiation, the absorption spectrum indicated the aggregation of Au(0) in absence of any stabilizer, shown by the high absorption baseline and disappearance of the broad SPR peak (Figure 1A). The TEM images collected from the unstable Au(I)-water system showed the expected aggregation of Au(0) nanostructures (Figure 1A’’). UV/VIS–NIR absorption spectra, daylight images, and TEM images of Au(I) in aqueous media confirmed that the Au(Me_2_S)Cl can undergo photochemical reduction to form metallic gold(0) without any external reducing agents. The evidence in Figure 1, supported our hypothesis for the rest of the manuscript. We deduced this hypothesis based on both the literature as well as our earlier AuNP work, which revealed that dimethyl sulfide (Me_2_S) is an ideal ligand for making nontoxic [55] gold nanoparticles, because of its inherent low toxicity and high volatility (e.g., it is responsible for the foul smell upon boiling cabbage). The literature notes that AuNP formation from Au(I) precursors is rarely explored in organic media [49] and completely unexplored in aqueous media, possibly because Au(I) compounds are known to undergo spontaneous decomposition in aqueous media even at ambient temperature, as confirmed in our current body of work in Figure 1.

### 3.2. In Situ Formation of Spherical Gold Nanoparticles within PNIPAm Microgels

To start with, the formation of spherical AuNPs was investigated under ambient and photoirradiation conditions within the PNIPAm-*co*-allylamine microgels (Figure 1B,C and Figure S2). Except for PNIPAm microgels, no additional or assisting reducing agents were employed during the reaction. AuNPs formed under photoirradiation exhibited smaller size, narrower size distribution, and higher photochemical quantum yield compared to those prepared under ambient conditions (Figure 1B vs. Figure 1C). Under both conditions, a typical PNIPAm-*co*-allylamine microgel sample containing Au(I) changes from colorless to pink color as evidenced by the evolution of an SPR peak around 575 nm (ambient conditions) or 530 nm (photoirradiation conditions) during the first 60 or 15 min of the reaction, respectively (Figure 1B,C). Under otherwise identical reaction conditions, the photoirradiated sample attained an arbitrary absorbance of 1.0 unit in less than 40 min, whereas the sample under ambient conditions required over 105 min to attain the same absorbance. The SPR peak maxima and full-width-half-max (FWHM) of the AuNPs spectra show that the AuNPs in the photoirradiated sample were monodispersed and smaller than those produced in ambient light (12.1 ± 4 nm or 862 cm^−1^ for λ_max_ = 530 nm vs. 21.0 ± 6 nm or 1270 cm^−1^ for λ_max_ = 575 nm, respectively). Additionally, FE-SEM images from both samples show visible differentiation of AuNPs and polymer microgel boundaries, with single to multiple AuNPs within a single microgel particle. Aggregated gold cores were more prevalent in the sample prepared using ambient light, as expected from the comparatively broad SPR peak. FE-SEM images indicate that in both cases, the microgels acted as host matrices which stabilized the AuNPs. Note that microgels containing multiple gold cores were more prevalent in the sample prepared under ambient conditions. PNIPAm microgels copolymerized with acrylic acid were synthesized separately to investigate the effect of surface charge of microgels during the formation of AuNPs. The surface charge of control PNIPAm-*co*-allylamine and PNIPAm-*co*-acrylic acid microgels were confirmed to be + 30 ±4.5 and − 25 ± 3.8 mV, respectively, as determined via zeta potential measurements. Except for a few minor variations, similar size, spherical AuNPs were obtained by utilizing negatively charged PNIPAm-*co*-acrylic acid gels, as shown in Figure 1D and Figure S3. Under similar experimental conditions, the acid-containing microgels attained 1.0 unit of arbitrary absorbance in less than 10 min, compared to 30 min for positively charged microgels (Figure 1C and Figure S3). The data from Table 1 showed that negatively charged microgels are preferable for making spherical AuNPs compared to positively charged PNIPAm microgels. The reason for this is likely due to the stronger electrostatic interactions between the negatively charged stabilizing surface and the Au^+^ atomic cations that potentially cover the surface of the AuNP; even in absence of such cations, the electrophilic nature of even neutral Au or other metal atoms is conducive to greater affinity to negatively charged stabilizers. The data from Figure 1 demonstrated the formation of spherical AuNPs within both positively charged and negatively charged PNIPAm hydrogel microspheres under similar experimental conditions. During these reactions, we have noticed that, under similar experimental conditions, usage of HAuCl_4_ does not result in the formation of AuNPs. No change in color or evolution of SPR was noticed even after 1 h of irradiation, emphasizing the significance of Au(I) precursor as a preferential replacement for HAuCl_4_ as the gold precursor in this study.

### 3.3. Hybrid Crystalline PNIPAm Microgels Containing Gold Nanoparticles

**Figure 2:**
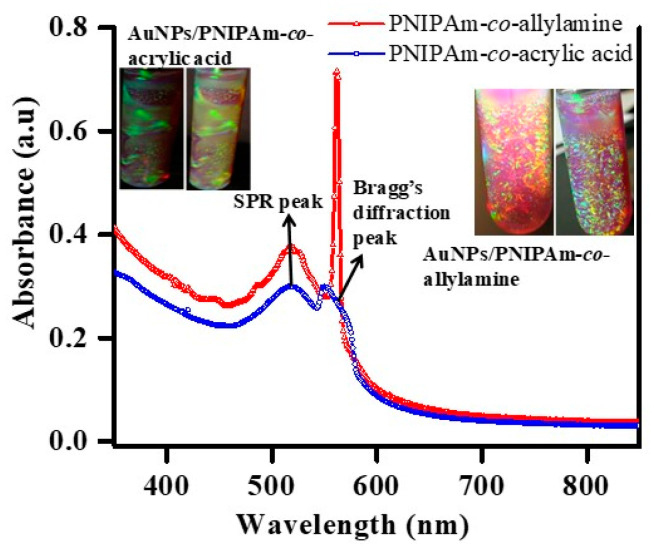
UV/VIS absorption spectra of hybrid poly(*N*-isopropylacrylamide) (PNIPAm) colloidal microgel crystals. The absorption spectra show the characteristic surface plasmon resonance (SPR) of spherical AuNPs and Bragg’s diffraction peak of orderly arranged PNIPAm colloidal crystals.

Spherical PNIPAm microgel nanoparticles are well-known for their ability to form environmentally sensitive colloidal crystals. When microgels containing AuNPs were concentrated (2–3 wt%) and centrifuged, followed by heating above their LCST and slow cooling, lustrous colloidal crystals were formed (Figure 2). Turbidity measurements of these hybrid colloidal crystals showed two characteristic peaks: a Bragg’s diffraction peak due to the orderly arrangement of PNIPAm colloidal crystals [4] and a second peak from the AuNPs’ SPR. The presence of the Bragg’s diffraction peak and SPR peak in the hybrid colloidal array signifies the retention of characteristic traits of both AuNPs and an orderly arrangement of PNIPAm crystalline microgels. Consistent with our investigation, there are reports of AuNPs formation within PNIPAm microgels and also demonstrations of color tunability within environmentally sensitive hydrogels and polymers [44]; however, the formation of hybrid PNIPAm colloidal crystals is demonstrated herein for the first time, to our knowledge. We believe that the additional environmentally sensitive SPR trait of these hybrid colloidal crystals will further benefit their photonic applications.

### 3.4. In Situ Formation of NIR-Absorbing Anisotropic Gold Nanomosaic (NIRAuNM) within PNIPAm-Co-Allylamine Microgels

**Figure 3:**
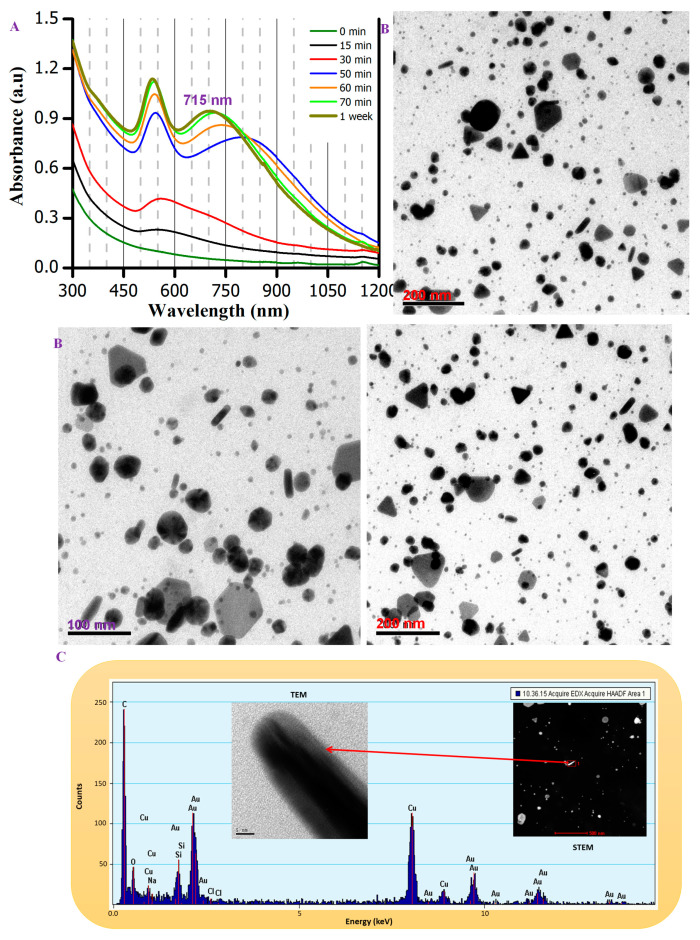
Characterization of NIR-absorbing anisotropic gold nanomosaic (NIRAuNM) formed within 0.5% wt/v of PNIPAm-*co*-allylamine microgels. In this specific reaction, photolysis was used to reduce 0.023 moles of Au(Me_2_S)Cl in the aqueous microgels medium by a single-step method. (**A**) Time-dependent absorption spectra, (**B**) TEM images, and (**C**) EDS data with TEM and scanning transmission electron microscope (STEM) images. The STEM image in the inset is collected using a high-angle annular dark-filed (HAADF) detector.

After investigating the formation of spherical AuNPs, our focus shifted towards the formation of more challenging and intriguing anisotropic and/or NIR-absorbing AuNPs. Particles which absorb in the NIR region open a new range of applications, most importantly plasmonic photothermal therapy. The photothermal heating of nanoparticles is shown to exhibit cell death through membrane blebbing [56]. Currently, there are scant literature examples of single-step methods for making NIR-absorbing anisotropic AuNPs and no literature methods that have demonstrated the formation of NIRAuNPs from Au(I) systems. Based on spherical AuNPs results, the light was selected as the energy source for exploring the formation of NIRAuNPs within PNIPAm-*co*-allylamine microgels. The entire procedure is performed using only two reagents: Au(Me_2_S)Cl and dialyzed PNIPAM-*co*-allylamine microgels. In a typical reaction, the formation of anisotropic AuNPs can be followed by observing the changes in time-dependent UV/VIS–NIR spectra (Figure 3). At the beginning of the irradiation, no NIR peaks were observed from the reaction mixture. The relatively high baseline in the UV region was attributed to microgels scattering. After 15–20 min of irradiation, the evolution of a broad SPR peak at 550 nm indicated the slow generation of spherical AuNPs. As the irradiation continued, the absorption band at 550 nm remained unchanged, whereas the evolution of a broad SPR peak in the NIR region with a peak maximum > 750 nm was observed. Upon further irradiation, the absorbance of the broad NIR peak (>750 nm) increased, accompanied by a blue shift in the peak maximum. After 70 min of continuous irradiation, the growth of the NIR peak halted with a peak maximum stabilizing at 715 nm. The time-dependent absorption spectrum (Figure 3) indicated that small particles bearing an SPR peak 550 nm were formed first, and we assumed eventual coalesce into anisotropic structures upon further irradiation. This was indicated by the evolution and continuous growth of the NIR peak but not the 550 nm peak. These data suggested a fusion growth pattern, where small particles congealed to form larger particles. To understand if the formation of these particles is only short-lived, TEM images were collected from the samples 1 week after synthesis. The absorption spectra obtained before the collection of TEM images showed no change in absorption spectra 1 week after synthesis, indicating the stability of the anisotropic nanoparticles. The mixture of different sizes and shapes of anisotropic particles observed in the TEM images were congruent with the broad NIR SPR peak. The mixture of shapes included rods, triangles/prisms, and other polygonal structures along with some large spherical/semispherical nanostructures, somewhat akin to the construction of a mosaic from individual tesserae blocks that need not be of a uniform shape. Hence, we coin the phrase “nanomosaic” for this type of particle in general; indeed, when we highlight the plasmonic absorption properties we will be using the more specific term “NIR-absorbing anisotropic gold nanomosaic” abbreviated henceforth as “NIRAuNM.” A closer observation reveals that the longitudinal length of these anisotropic nanostructures, especially rods and triangles, was over 50 nm, which was consistent with both their NIR SPR peak (λ_max_ > 700 nm) and published literature [31,37,57]. We believe that the blue shift in the SPR during irradiation is likely due to the reversible conversion of initial larger-sized anisotropic shapes to smaller anisotropic shapes or partial conversion to spherical shapes caused by continuous irradiation. EDS spectra revealed the chemical composition of these NIRAuNMs (Figure 3). Au peak positions observed in the EDS spectrum are in good agreement with literature results [57,58]. As the NIRAuNM precursor contains sulfur, the absence of any sulfur signal in the EDS spectrum confirms that the precursor ligand has been driven out of the system and thus the particles are purely Au-based. Signals from C, O, Cu, and Si were due to the PNIPAm stabilizer and Cu grid used in the EDS analysis. Au peaks confirmed the elemental composition and purity of the nanoparticles. Figure 3 confirms the formation of stable NIR-absorbing anisotropic shapes within PNIPAm-*co*-allylamine microgels without the aid of any exogenous reducing or growth-assisting agents. To the best of our knowledge, this is the simple, straight-forward, and single-step synthetic technique for making a mixture of stable, NIRAuNMs within a/microgel medium.

**Figure 4:**
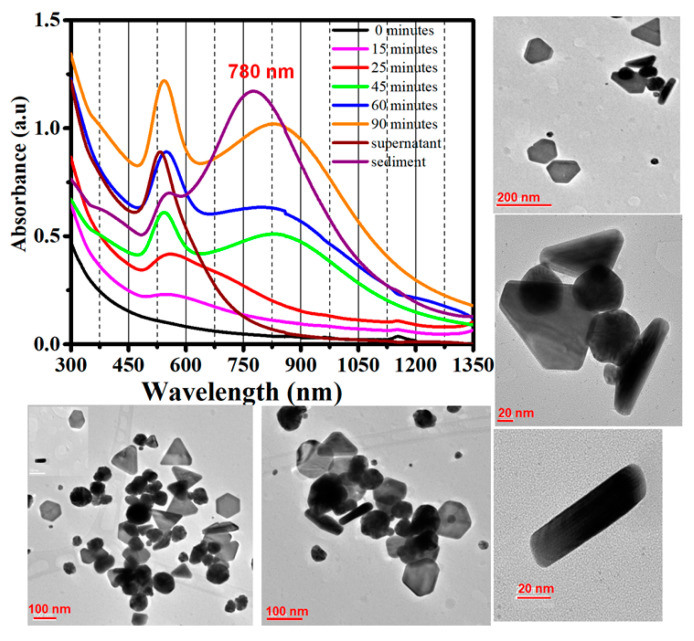
Characterization of NIRAuNM formed within 0.5% wt/v of PNIPAm-*co*-allylamine microgels by photolysis of 0.033 mmol of Au(Me_2_S)Cl in the aqueous microgel medium by a single-step method. Time-dependent absorption spectra and TEM images are included.

To understand the effect of Au(I) concentration on the tunability of the NIR SPR, the reaction was repeated at two different Au(Me_2_S)Cl concentrations other than the one described in Figure 3. Figure 4 shows the formation of NIRAuNM at a higher concentration compared to the sample in Figure 3. The time-dependent absorption spectra in Figure 4 are like those noticed in Figure 3. Specifically, both figures show an initial evolution of a visible absorption peak, followed by the evolution of a NIR peak, followed by a red-shift in the NIR peak maxima with continuous irradiation, culminating with an SPR peak in the NIR region (λ_max_ > 650 nm). At the higher concentration of Au(Me_2_S)Cl, the sample exhibited a red-shifted NIR absorption peak maximum at around 825 nm compared to the 715 nm NIR peak noticed in the sample with a lower concentration of Au(Me_2_S)Cl. The two samples established the correlation between Au(I) concentration and the size of the particles, consistent with their SPR spectra. At lower concentrations of Au(I), the NIRAuNMs exhibited a NIR peak maximum around 715 nm, whereas the higher Au(I) concentration resulted in a red-shifted NIRAuNM with a peak maximum at 825 nm. Additionally, comparing the TEM images in Figure 3 and Figure 4 showed that the sample containing the higher concentration of Au(I) contained larger anisotropic shapes with longitudinal lengths over 100 nm for multiple particles compared to the prevalent 50 nm sizes at the lower Au(I) concentration. Evident from Figure 3, at the reaction’s endpoint, the sample did not contain a dominant NIR peak. Additionally, absorption spectra from Figure 4 showed the successful enrichment of NIR absorbance by benchtop centrifugation. The sediment sample exhibited a dominant NIR peak compared to uncentrifuged samples.

**Figure 5:**
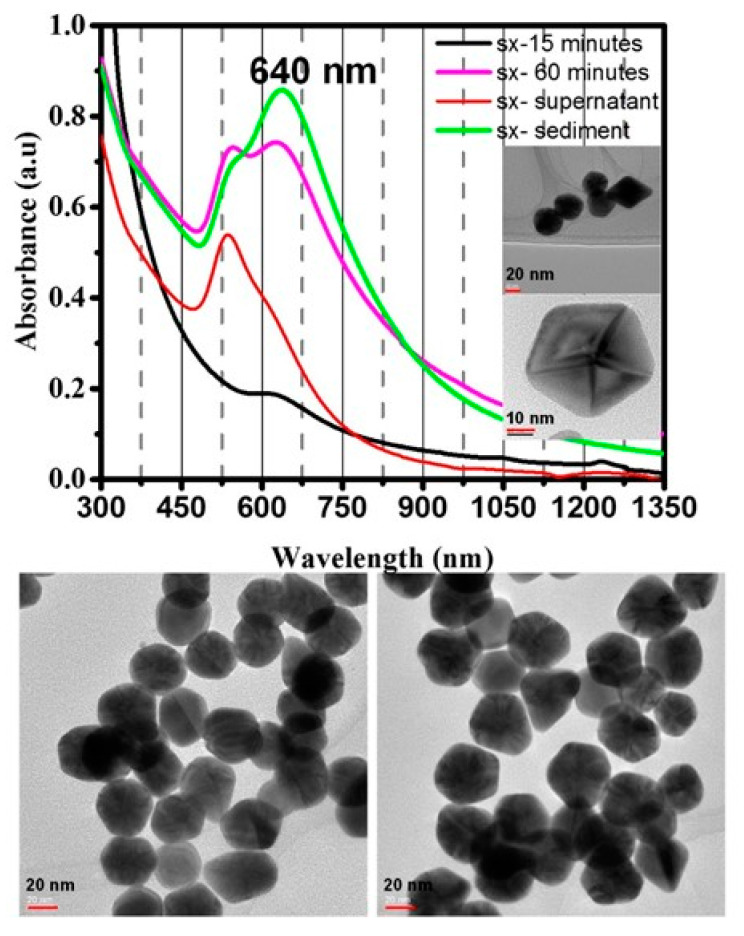
Characterization of NIRAuNM formed within 0.5% wt/v of PNIPAm-co-allylamine microgels by photolysis of 0.016 mmol of Au(Me_2_S)Cl in aqueous microgels media by a single-step method. Time-dependent absorption spectra and TEM images are included.

As expected, a sample containing a lower content (0.016 mmol) of Au(I) compared to both previous samples exhibited a blue-shifted NIR absorption peak (Figure 5). Each TEM image, again even for this lower-Au-content composition, comprised a nanomosaic without a dominant geometry for the tesserae elements that comprised the mosaic with interesting polygonal shapes with an average size of 30 ± 7 nm for each tessera in this lower-Au-content sample–in contrast to the nanorods and nanotriangles that comprised the tesserae observed in the previous samples with greater Au content. This is again akin to the mosaic analogy that different mosaics have their elements of beauty although the collection of tesserae is different both within the same mosaic and between different mosaics. Still, it is often the case that the tesserae in the same mosaic exhibit greater similarity within one another (i.e., an assortment of polygonal shapes in Figure 5 vs. an assortment of nanorods and nanoprisms in Figure 3 and Figure 4). Concluding from the absorption data and TEM images obtained from three different samples with varying concentrations of Au(I) precursor, it is clear that the size and SPR of anisotropic NIRAuNM are dependent on Au(I) concentration, higher Au(I) concentration resulted in larger and red-shifted NIRAuNM. UV/VIS/NIR and TEM data of different Au(I) concentration samples imply that photoirradiation initially results in the formation of spherical AuNPs that eventually grow into larger anisotropic structures that remain unaltered after their formation. Based on the existing literature overviewed above, we determined that it was a priority objective to investigate the feasibility of making NIR-absorbing anisotropic AuNPs by use of an Au(I) precursor and to capture the complete reaction in a single-step method that is absent of CTAB and other growth or reducing agents. Based upon the UV/VIS/NIR spectra, TEM images, and EDS spectra presented herein for the samples discussed, we believe we have demonstrated the achievement of this objective.

Due to its low stability and sensitivity to water and temperature, we conjectured that Au(Me_2_S)Cl could be reduced to Au(0) even under other experimental conditions besides photolysis. Using our single-step methodology, the synthesis of NIRAuNM containing mixed anisotropic shapes was also found to be driven by thermolysis and sonolysis conditions (Figures S4–S6). Based on the absorption spectra as noted in Figure S4, the thermolysis procedure emerged as a more facile and promising process compared to photoirradiation for making strong NIR-absorbing anisotropic AuNPs. Samples subjected to thermolysis exhibited the NIR peak as their major peak even without requiring centrifugation. However, for yet unidentified reasons, TEM images (Figures S4 and S5) revealed large, polyhedral nanostructures along with hitherto unreported tailed structures. EDS (Figure S5) showed no traces of contamination or sulfur, confirming the structures to be pure AuNPs. Particles prepared by the sonolysis process also exhibited a mixture of shapes, akin to the nanotesserae observed by photolysis (Figures S4 and S6). These results indicate that under the appropriate conditions, i.e., light, thermal, and kinetic energy, one can reduce the Au(Me_2_S)Cl precursor to Au(0), and when this precursor is reduced in an appropriate stabilizing medium, spherical or anisotropic AuNPs are formed.

**Figure 6:**
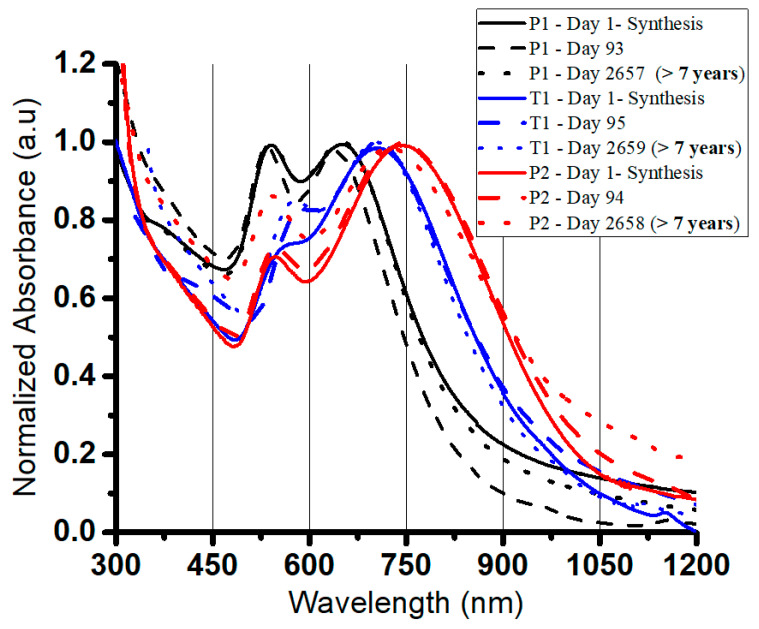
Demonstration of extreme time-dependent stability of NIRAuNM stabilized within PNIPAm-*co*-allylamine microgels. P1, T1, and P2 refer to NIRAuNM samples prepared by photochemistry, heating, and photochemistry, respectively, within PNIPAM-*co*-allylamine microgels media.

Evaluating the shelf-life stability of AuNPs is crucial in establishing our method’s viability for producing AuNPs toward commercial and/or clinical applications. Samples with different Au(Me_2_S)Cl concentrations were synthesized within PNIPAm-*co*-allylamine microgels by photoirradiation and heating. Long-term stability was evaluated by directly comparing their absorption spectra at different time intervals. All tested samples were stored under ambient conditions. Figure 6 shows changes in absorption spectra for NIR-absorbing AuNP samples. Selected samples were planned to be tested for shelf-life stability after 3 months of storage under ambient conditions; however, after noticing their unexpected, unchanged absorption profile, samples were placed into ambient storage, and unintentionally left there for 7 years, at which time, they underwent additional testing. Overall, the absorption spectra in Figure 6 showed no changes between days 1 (solid line) vs. day 90 (dashed line), indicating that these samples did not undergo any chemical or physical changes in composition or AuNP morphology (i.e., these data suggested that the samples exhibited no decomposition or aggregation tendency after the first 90 days of storage). Incredibly, the negligible changes that were noticed after 7 years of ambient storage indicate their strong commercial utility. Only two minor changes were observed. The first was an increase in the visible absorption peak, and the second was a rise in the baseline absorbance at wavelength ≥1000 nm (dotted lines, Figure 6). We assume these minor changes could be due to the rather slow conversion of minute amounts of anisotropic particles to spherical particles. Future work will involve analysis following NIST stability testing protocols to estimate the shelf life of these highly stable NIRAuNM. We believe the extraordinary shelf-life stability of the NIRAuNM synthesized using the single-step method, containing no CTAB or any additional reducing agents, merits fine-tuning the reaction parameters. This fine-tuning will define the synthesis conditions necessary to control the shape- and size-selective NIR-absorbing anisotropic AuNPs, while utilizing the highly desired, mild chemical environment as opposed to the harsh surfactants and reducing agents.

### 3.5. Demonstrating Broad Applications by Altering the Polymer Stabilizer

**Figure 7:**
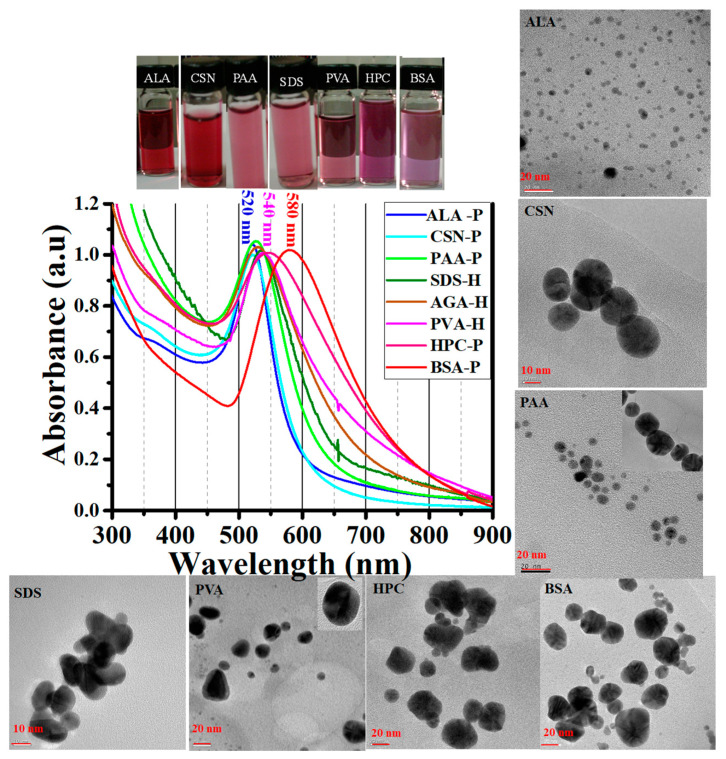
Characterization of spherical AuNPs stabilized in different polymers under photoirradiation (P) and heating (H) by daylight images, UV/VIS absorption spectra, and TEM images. ALA = alginic acid; CSN = chitosan; PAA = polyacrylic acid; SDS = sodium dodecyl sulfate; PVA = polyvinyl alcohol; HPC = hydroxypropyl cellulose; BSA = bovine serum albumin.

Encapsulating AuNPs within polymer matrices was found to be an effective way to enhance the stability and functions of metal nanoparticles. Conjugation of polymers to AuNPs and/or using them as templates allows one to build structural and functional units useful for many optoelectronic and biological applications [59]. We tested the feasibility, versatility, and suitability of our methodology for in situ formation of stable AuNPs within various polymers, surfactants, and biological media. Stabilizing matrices were selected based on chemistry and application significance, i.e., CS was selected for its biocompatibility, SDS for its surfactant chemistry, alginic acid for its acid (–COOH) chemistry, and HPC and PVA for their hydroxy (–OH) chemistry. The pH-sensitive PAA was selected for its broad usage in household/personal care products, moisturizers, and super porous microgels. The binding and coating capabilities of PVA are efficiently utilized in the food industry [60]. Agar, the gelatinous polysaccharide which solidifies at 32–40 °C is a crucial component in many biological assays [61]. HPC is a derivative of cellulose, which is soluble in water as well as many organic solvents, is typically used in tablet binding, tablet coating, and as an ophthalmic protectant and lubricant [62]. As one of the most important nutrients in cell culture studies, BSA [63] is a crucial protein in various biological studies. In the existing literature, a few reports exist for making AuNPs using the above stabilizing media [63,64,65,66,67,68]. Figure 7 illustrates the formation of spherical AuNPs within these stabilizers by photoirradiation or by heating. For yet unidentified reasons, some polymers have exhibited the formation of AuNPs by photoirradiation, while others have formed AuNPs through heating. Table 2 summarizes the optical and morphological properties of these different AuNPs. TEM images revealed some anisotropic structures, which contrasted with the single SPR peak. The formation of nonspherical particles in some of these media is hypothesized to be polymer- and Au(I)-concentration-dependent, which is currently under investigation. Visible colors, the evolution of SPR peaks, and TEM images confirmed the formation of AuNPs regardless of the stabilizer or energy source and using Au(Me_2_S)Cl as the precursor without additional reagents. TEM images showed AuNPs with sizes ranging from 5 to 50 nm formed within these stabilizers. From the UV–VIS and TEM data, the relation between the size of the particles and the chemistry of the stabilizers was not fully established; however, we observed that polymers with a negative surface charge tend to stabilize AuNPs with blue-shifted, narrow SPR peaks and exhibit narrowly dispersed particle sizes. The exception to this observation was the CSN polymer for reasons not yet understood. Alginic acid-stabilized samples showed a narrow SPR peak at 525 nm with TEM images for the same samples revealing particles with sizes ranging between 5 and 10 nm. Samples stabilized in SDS, a negatively charged surfactant, also exhibited narrow SPR with a peak maximum at 528 nm and particle size ranging from 5 to 10 nm. In contrast, HPC and BSA formed AuNPs that exhibited red-shifted, broader SPR peaks, with particle sizes ranging between 30 and 40 nm. Under similar experimental conditions, we have not noticed any formation of AuNPs within these media using HAuCl_4_ as a precursor without the aid of additional chemical reagents. By demonstrating the formation of AuNPs within this wide variety of media, the extensive applicability of using Au(Me_2_S)Cl as a precursor for making spherical AuNPs without any reducing or assisting chemical reagents is validated. This demonstration will provide flexibility for nanoparticle researchers to select and grow AuNPs within desired stabilizing media based on their requirements and application.

### 3.6. Photothermally Driven Release of a Model Dye from NIRAuNM Containing PNIPAm–Co-Allylamine (Hybrid) Microgels

The ability of metallic nanoparticles to transduce absorbed light into heat has been used for direct cell killing and drug delivery applications [10,19,56,57]. For drug delivery and PPTT, plasmonic nanoparticles or dyes which absorb in the NIR region are considered superior to visible-absorbing NPs for numerous reasons [12,14,57]. Thermoresponsive hydrogel microparticles, which exhibit volume-phase transitions, particularly photothermally modulated phase or size transitions induced by guest metallic nanoparticles are also known for their potential use in “smart,” “switchable” devices [45,69,70,71]. To the best of our knowledge, until now only two groups have demonstrated a volume-phase transition in PNIPAm particles using NIR-sensitive AuNPs, the Halas and West group, who demonstrated photothermal phase transition and drug delivery using Au nanoshells, and the Kumacheva group, who demonstrated the photothermally driven swelling–shrinking ability of PNIPAm microgels by loading CTAB-stabilized AuNRs [70,72]. The major distinction and advantage of our NIRAuNMs are that they exhibit a large absorption cross-section (700–1200 nm) that facilitates the use of inexpensive white light/broad-band lamp sources instead of expensive, wavelength-specific monochromatic lasers for exciting the surface plasmon electrons.

**Figure 8:**
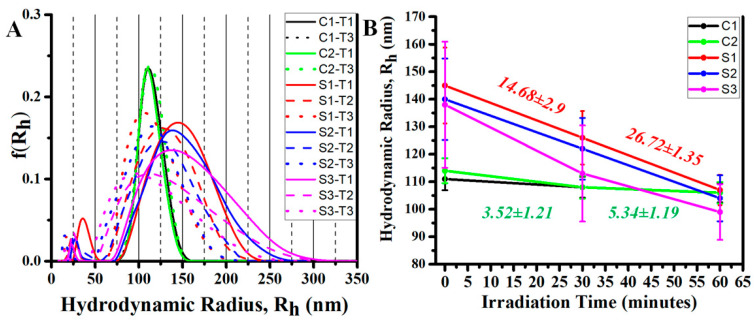
Demonstration of size shrinkage hybrid PNIPAm-*co*-allylamine microgels containing NIR-absorbing AuNM (hybrid microgels) on irradiation with NIR light source. (**A**) Light scattering data representing the changes in hydrodynamic radius of PNIPAm-*co*-allylamine microgels containing NIRAuNMs (S1–S3) vs. control microgels containing no AuNPs (C1–C2). S–irradiation cycle and T–irradiation time (0, 30, and 60 min). Control microgels data for two cycles of irradiation, whereas hybrid microgels are exposed to three cycles of irradiation. (**B**) Changes in hydrodynamic radius (Rh) of the hybrid microgels vs. irradiation time. C and S represent control and hybrid microgels, respectively, and 1, 2, and 3 represent cycles of irradiation.

**Figure 9:**
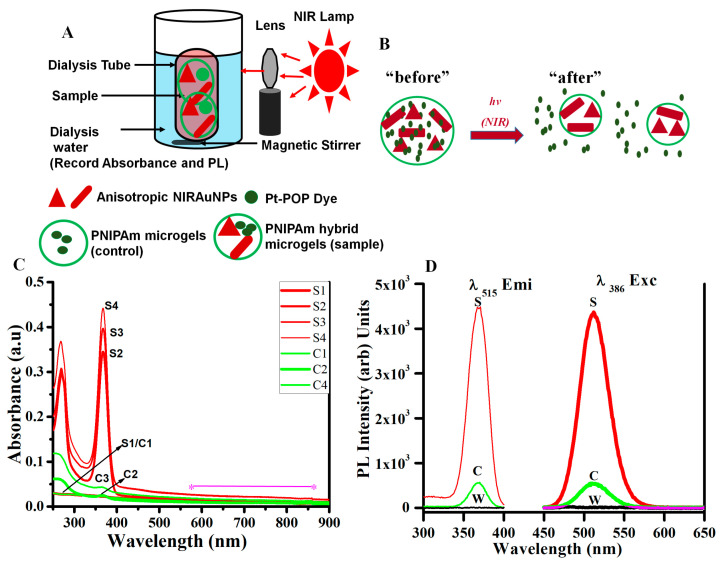
Demonstration of the release of a dye molecule from hybrid PNIPAm-*co*-allylamine microgels containing NIR-absorbing AuNM (hybrid microgels) on irradiation with NIR light source. (**A**) Schematic illustration of dye-release studies from the hybrid microgels containing NIRAuNMs and Pt-pop dye. (**B**) Schematics of dye release “before” and “after” irradiation with the NIR light source. (**C**) Changes in absorption spectra at 0, 15, 30, and 45 min (1,2, 3, and 4) of irradiation for control and hybrid microgels. (**D**) Photoluminescence spectra of dialysis water at 45 min of irradiation (solid lines: emission spectra and dashed lines: excitation spectra). W–blank water, C–control microgel, and S–hybrid microgel.

Herein, in situ stabilized, CTAB-free NIRAuNMs were employed in a proof-of-concept experiment, which demonstrated their ability to release a molecular dye (PtPOP) based on photothermally driven “shrinking” of PNIPAm-*co*-allylamine microgel particles. A control microgel containing no AuNPs was tested under identical experimental conditions. The control and hybrid microgels showed similar phase transition temperatures (32 °C, Figure S1). Based on LCST data, the microgel samples were maintained at 32 °C using circulating water flow. A 100-W quartz tungsten halogen lamp was selected as the light source. The irradiance curve for the lamp is shown in Figure S7. Lamp output under experimental conditions was measured using a light-output meter and was determined to be 0.13 W/cm^2^. A converging lens and a 500 nm cutoff filter were used to focus the radiation from the lamp onto the sample vial and to eliminate any photothermal effect of higher energy, i.e., UV/VIS absorption of the NIRAuNM sample on photothermal studies. The experiments were conducted by placing the sample test tube in the sample holder of a dynamic light scattering instrument (AVL-5000, Langen, Germany, ) followed by irradiation with the NIR lamp. The constant water flow helped to maintain the sample temperature uniformly at 32 °C. Heating cycles were separated by 1-hour intervals to allow the samples to cool to ambient temperature for deswelling. The UV/VIS/NIR spectra of hybrid microgel samples used for both volume-size change and dye release studies are shown in Figure S8. Figure 8 shows the changes in the average hydrodynamic radius of hybrid and control microgel particles upon irradiation with a NIR lamp source. The greater decrease in size of PNIPAm microgels containing NIRAuNMs relative to the control gel particles containing no AuNPs indicated PPTT activity of NIRAuNMs. Microgel particles containing gold nanoparticles exhibited an average decrease of 14.6 ± 2.9 nm and 14.7 ± 1.5 nm in hydrodynamic radius (R_h_) after the first 30 min and after the second 30 min of exposure, respectively, for three cycles. At the end of 60 min, the total decrease in the size of the hybrid microgels was found to be 26.72 ± 1.35 nm accounting for 3 cycles. In contrast, the unloaded control microgels exhibited a marginal decrease of 3.5 ± 1.2 nm and 5.3 ± 1.2 nm in R_h_ after 2 cycles of irradiation, under identical experimental conditions. This order of magnitude difference in R_h_ changes of hybrid microgels compared to control microgels demonstrates the photothermal activity of NIRAuNMs. Further validation of photothermal behavior was demonstrated by the release of a molecular dye, (K_4_[Pt_2_(P_2_O_5_H_2_)_4_]·2H_2_O) (also known as Pt-POP) from hybrid microgels upon NIR irradiation (Figure 9). Retention of the NIRAuNMs and the dye (Pt-POP) within hybrid microgels after stirring and centrifugation was verified by UV/VIS/NIR spectra (Figure S8). UV/VIS/NIR spectra of hybrid and control microgels after loading Pt-POP exhibited a characteristic UV transition band at 380 nm (Figure S8) with similar absorbance, indicating the presence of a similar concentration of the dye in both hybrid and control microgel particles. For demonstrating photothermal release, both the microgel samples were enclosed in 3000 Da dialysis tubing and then subjected to irradiation by maintaining the temperature at 32 °C. Dye release from microgel particles was monitored by measuring the changes in UV–VIS absorption and photoluminescence of the water sample in which the dialysis tube was immersed (schematic in Figure 9A,B). Initially, both samples exhibited an identical baseline and no absorption at 367 nm. After 30 and 45 min of irradiation, the percentage variation in absorbance at 367 nm from hybrid microgel samples was 165% and 164%, respectively. This is a clear indication of Pt-POP dye release from hybrid microgel particles in contrast to an insignificant release of dye from the control microgel particles (Figure 9C). In the evaluation of individual absorbance changes at 367 nm for control and hybrid samples, the absorbance of the hybrid sample changed from 0.0225 to 0.4415 (arbitrary units), whereas the absorbance of the control microgels changed marginally from 0.0225 to 0.0431 in 45 min of irradiation, indicating an order of magnitude difference in absorbance changes for hybrid microgels compared to control microgels. It can be noticed from the absorbance changes (Figure 9) that potentially, most of the dye was released within the first 15 min. As Pt-POP exhibited strong green emission, a strong difference in photoluminescence emission intensity was also noticed from hybrid vs. control microgels (Figure 9D). There is an order of magnitude (or about 90%) variation in emission intensity between hybrid and control microgels, like absorbance changes. These dye release data not only demonstrate the release of the dye molecule (Pt-POP) due to photothermally driven size changes but also reconfirm photothermally driven shrinking effect in hybrid PNIPAm microgels due to the presence of NIRAuNMs. Light-activated therapies to kill diseased cells and tissues in a noninvasive manner using exogenous agents with large absorption cross-section are well known [56]; however, most demonstrations have been performed using CTAB-stabilized AuNRs. In contrast, based on our ongoing cytotoxicity studies with CTAB-free anisotropic AuNPs, that have already demonstrated more than 50% lower cytotoxicity vs. CTAB-stabilized AuNRs, we strongly believe that our CTAB-free NIRAuNMs with a large absorption cross-section, demonstrating photothermally driven size changes and dye release, are expected to exhibit potential beneficial effects as photothermal agents for cell killing studies, once the cytotoxicity of our AuNPs is fully established. 

## 4. Conclusions

The present investigation demonstrates two important discoveries: (1) development and use of an unexplored methodology wherein an Au(I) complex serve as a gold precursor to generate AuNPs of different sizes and shapes (nanomosaics) in various stabilizing media and (2) the first-ever direct formation of NIR-absorbing AuNPs within thermoresponsive PNIPAm microgels. The facile wet photochemical method resulted in NIR-absorbing Au nanomosaic (NIRAuNM) of diverse anisotropic sizes and shapes and a corresponding wide absorption cross-section. No exogenous reducing agents are needed to reduce the gold precursor. The Au(Me_2_S)Cl complex, a commercially available well-known starting material for Au(I) complexes, has proven to be an effective precursor for making both spherical and anisotropic AuNPs by tuning experimental conditions. The instantaneous reaction of gold(I) sulfide in aqueous media is shown to be responsible for the reduction process without exogenous reducing agents. The flexibility of the reduction of Au(Me_2_S)Cl to form AuNPs within a wide variety of media is demonstrated. Even with a mixture of shapes to compose nanotesserae, a direct correlation is noticed between the size of anisotropic AuNPs on the one hand (longest dimension for representative tesserae of each sample) and the NIR SPR peak maximum on the other hand. Additionally, a strong direct correlation is observed between the precursor concentration and average longitudinal length and NIR SPR of the anisotropic structures. The higher concentration of Au(I) resulted in large size particles with red-shifted NIR absorption. The demonstration of photothermally driven shrinkage in size followed by the release of a dye from the hybrid microgels exhibits a proof-of-concept result toward potential drug release studies in the future, but for this, higher LCST slightly above body temperature is required. The proof-of-concept result, nonetheless, led to validation studies of the photothermal transduction property of NIRAuNM produced by our distinct one-step photochemical methodology. Based on our ongoing cytotoxicity tests and because these particles do not rely on CTAB or additional chemicals, they are expected to be far less toxic compared to CTAB-containing NIRAuNPs. Of consequence is our development of this method that uses conditions comparatively milder than the literature standard for the formation of different sizes and shapes of AuNPs. In this regard, we suggest our method will add to the existing “green” synthesis methods, and thus will have an impact on reducing the environmental footprint of these technologies. Finally, the preparation of specific drug delivery systems with a variety of model drugs is underway, taking advantage of the approach herein.

## Figures and Tables

**Table 1:** Correlating absorption spectra (SPR) with a TEM-determined particle size (average diameter, d_avg_) of gold nanoparticles (AuNPs) obtained from different compositions of poly(*N*-isopropylacrylamide) (PNIPAm) microgels.

Stabilizing Media	Experimental Condition	SPR λ_max_ (nm)	FWHM * (nm)	FWHM * (cm^−1^)	TEM d_avg_ (nm)	PQY *
PNIPAm-*co*-allylamine microgels	Ambient light	575	676	5825	21.0 ± 6.0	NA
PNIPAm-*co*-allylamine microgels	Photoirradiation	535	591	4584	12.1 ± 4.0	0.07
PNIPAm-*co*-acrylic acid microgels	Photoirradiation	533	595	4932	10.6 ± 3.7	0.1

* PQY = photochemical quantum yield; FWHM = full-width-half-max (nanometers (nm) and wavenumbers (cm^−1^)).

**Table 2:** Optical and morphological properties of AuNPs obtained under various experimental conditions within the wide range of stabilizing media (using Figure 7 data).

Stabilizing Media	Experimental Condition (Time, Minutes)	SPR ʎ_max_ * (nm)	FWHM * (nm)	FWHM * (cm^−1^)	TEM d_avg_ (nm)
Alginic acid (ALA)	Photoirradiation (15 min)	521	96	3680	4.1 ± 1.6
Chitosan (CSN)	Photoirradiation (45 min)	522	102	3883	19.8 ± 1.6
Polyacrylic acid (PAA)	Photoirradiation (15 min)	527	130	4864	6.5 ± 2.2
Sodium dodecyl sulfate (SDS)	Heating (10 min)	535	122	4222	Nonspherical, fused 20 ± 4
Polyvinyl alcohol (PVA)	Heating (20 min)	538	166	5772	Nonspherical, 25 ± 5
Hydroxypropyl cellulose (HPC)	Photoirradiation (30 min)	547	210	6764	Nonspherical, fused, 30 ± 5
Bovine serum albumin (BSA)	Photoirradiation (90 min)	580	178	5142	Nonspherical, 35 ± 5

* SPR = surface plasmon resonance, peak maxima; FWHM = full-width-half-max (nanometers (nm) and wavenumbers (cm^−1^)). The heating and photoirradiation time varied depending on the nature of the polymer stabilizer.

## Supplementary Materials

The following are available online at https://www.mdpi.com/2079-4991/10/7/1251/s1, Figure S1: Light scattering data representing the changes in hydrodynamic radius of PNIPAm-*co*-allylamine microgels containing NIRAuNMs vs. control microgels containing no AuNPs. Figure S2: Additional TEM and FE-SEM images of isotropic, spherical AuNPs stabilized within PNIPAm-*co*-allylamine microgels, produced by photolysis. Figure S3: Absorption spectra of AuNPs formed using Au(Me_2_S)Cl precursor using PNIPAm-*co*-acrylic acid microgels. The traces illustrate the changes in absorbance of AuNPs with respect to irradiation time. Figure S4: Characterization of NIRAuNM formed within 0.5% wt/v of PNIPAm-co-allylamine microgels by thermolysis (top) and sonolysis (bottom) of 0.033 mmol of Au(Me_2_S)Cl in aqueous microgel media by a single-step method. Absorption spectra and TEM micrographs are shown. Figure S5: TEM micrographs and EDS spectrum of anisotropic, polyhedral, tailed gold nanostructures obtained by thermolysis. Figure S6: Absorption spectra and TEM micrographs of anisotropic AuNPs obtained by sonolysis. Figure S7: Irradiance curve for quartz tungsten halogen (QTH) lamp (100 W) used as a radiation source to demonstrate photothermal volume phase transition and dye-release studies (taken from Newport website, model 6333). Figure S8: Absorption spectra of PNIPAm-*co*-allylamine microgel samples containing NIRAuNMs vs. control microgels containing no AuNPs.
