# Supplementary material for: Single-Step Photochemical Formation of Near-Infrared-Absorbing Gold Nanomosaic within PNIPAm Microgels: Candidates for Photothermal Drug Delivery
Source: Nanomaterials (Basel). 2020 Jun 28;10(7):1251. doi: 10.3390/nano10071251 (PMC7408514; doi:10.3390/nano10071251)
Supplement: Supplementary file 1 [file nanomaterials-10-01251-s001.pdf]

Article

Single-Step Photochemical Formation of Near-Infrared-Absorbing Gold Nanomosaic within PNIPAm Microgels: Candidates for Photothermal Drug Delivery

Sreekar Babu. Marpu [1,*], Brian Leon. Kamras [1,†], Nooshin MirzaNasiri [1,†], Oussama Elbjeirami [1,‡], Denise Perry. Simmons [2], Zhibing Hu [3,‡] and Mohammad A. Omary [1,*]

1 Department of Chemistry, University of North Texas, Denton, TX 76203, USA; briankamras@my.unt.edu (B.L.K.); nooshinmirzanasiri@my.unt.edu (N.M.); elbjeirami@unt.edu (Q.E.)
2 Department of Mechanical and Energy Engineering, University of North Texas, Denton, TX 76203, USA; denise.simmons@unthsc.edu
3 Department of Physics, University of North Texas, Denton, TX 76203, USA
* Correspondence: sreekarbabu.marpu@unt.edu (S.B.M); mohammad.omary@unt.edu (M.A.O)
† These authors contribute equally to this work.
‡ Deceased.

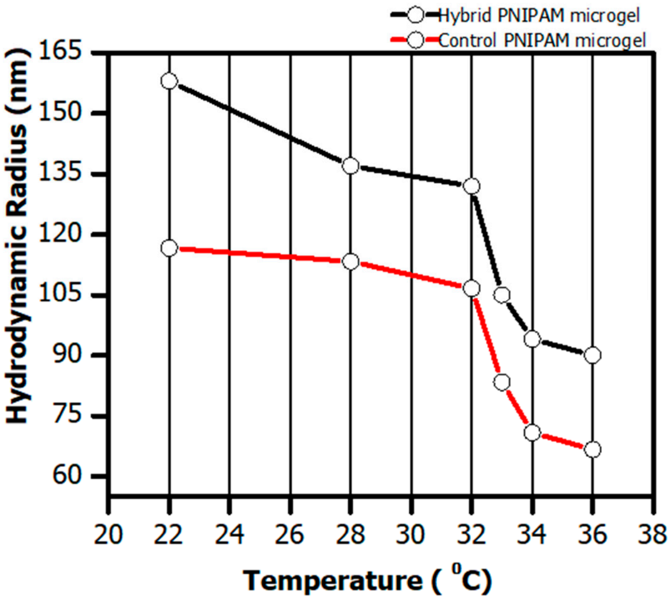

Figure S1: Light scattering data representing the changes in hydrodynamic radius of PNIPAm-*co*-allylamine microgels containing NIRAuNMs vs control microgels containing no AuNPs. The LCST is determined from this data and used for NIR exposure studies.

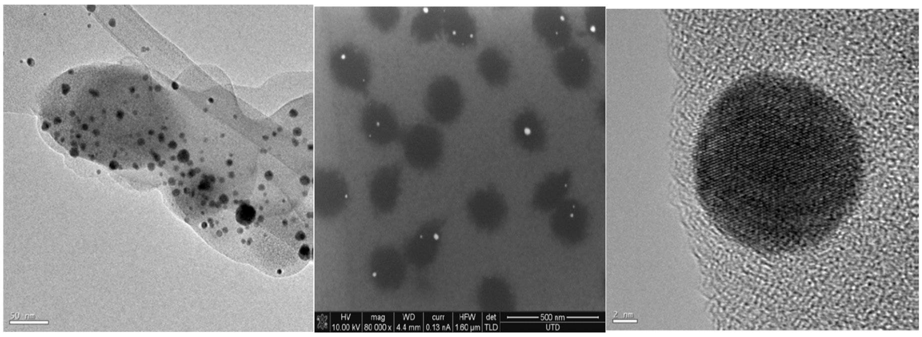

Figure S2: Additional TEM and FE-SEM images of spherical AuNPs stabilized within PNIPAm-*co*-allylamine microgels, produced by photoirradiation of Au(Me_2S)Cl.

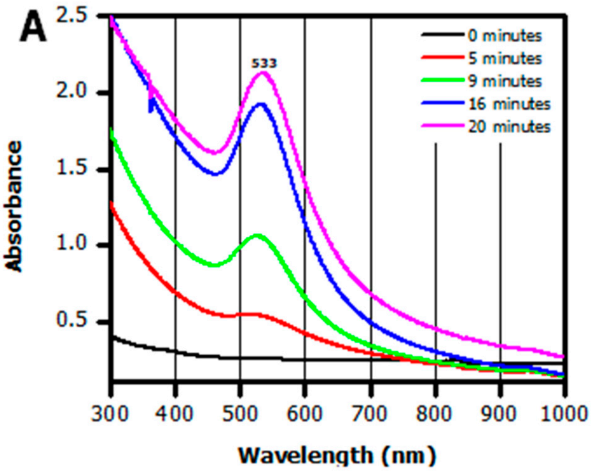

Figure S3: Absorption spectra of spherical AuNPs formed by photoirradiation using Au(Me_2S)Cl precursor within PNIPAm-*co*-acrylic acid microgels. The traces illustrate the changes in absorbance of AuNPs with respect to irradiation time.

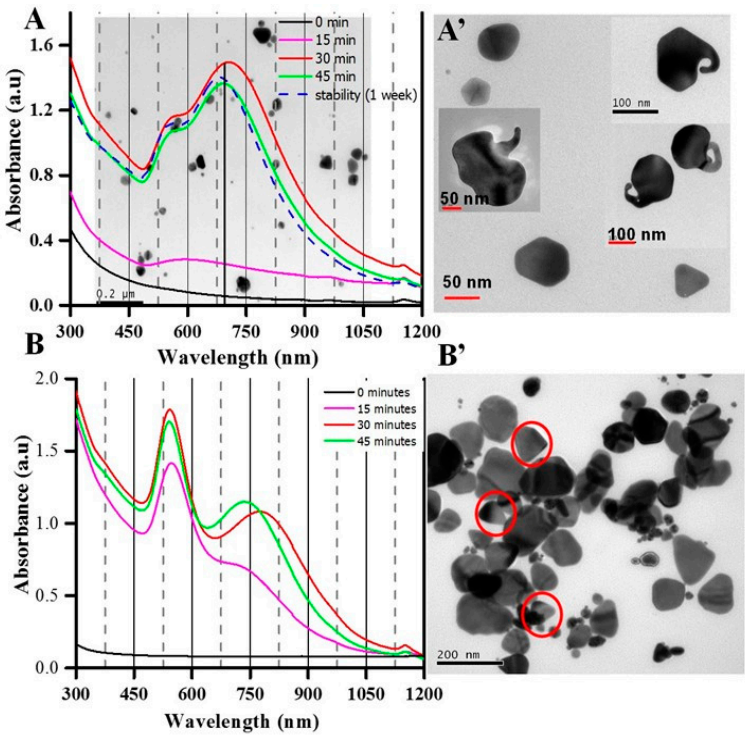

Figure S4. Characterization of NIRAuNM formed within 0.5% wt/v of PNIPAm-co-allylamine microgels by thermolysis (top) and sonolysis (bottom) of 0.033 mmol of Au(Me_2S)Cl in aqueous microgel media by a single-step method. Absorption spectra and TEM micrographs are shown.

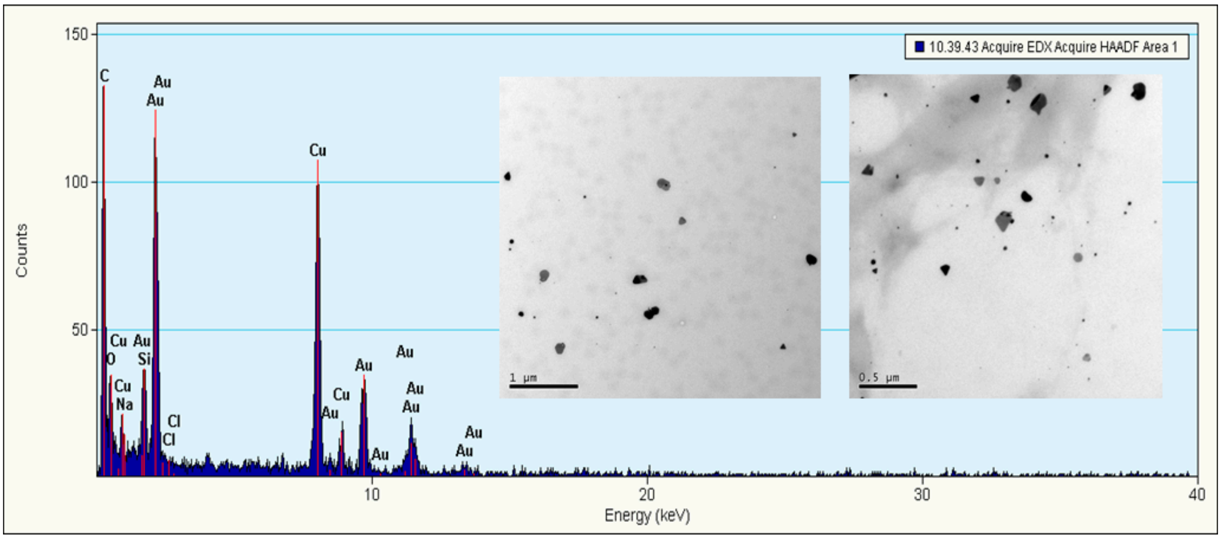

Figure S5: TEM micrographs and EDS spectrum of anisotropic, polyhedral, tailed gold nanostructures obtained by thermolysis.

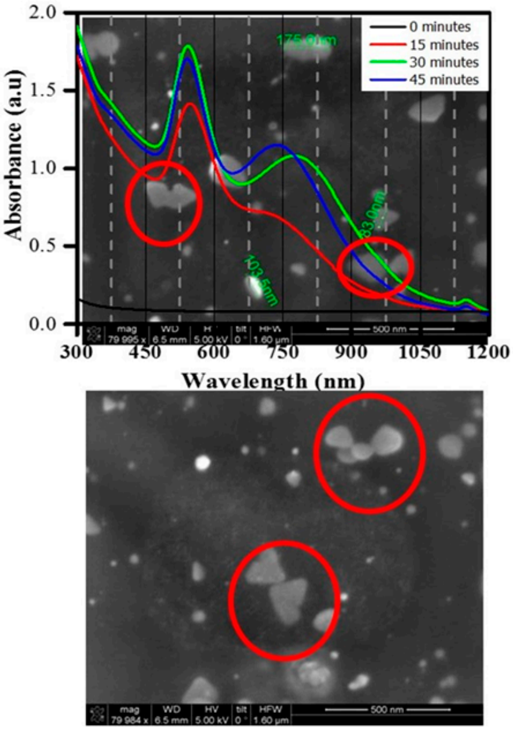

Figure S6: Absorption spectra and TEM micrographs of anisotropic AuNPs obtained by sonolysis.

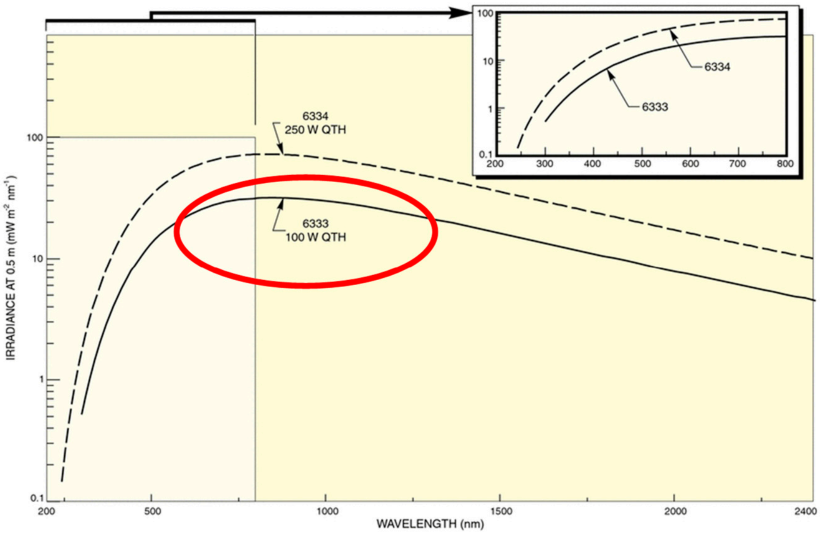

Figure S7: Irradiance curve for quartz tungsten halogen (QTH) lamp (100 W) used as a radiation source to demonstrate photothermal volume phase transition and dye release studies (taken from Newport website, model 6333).

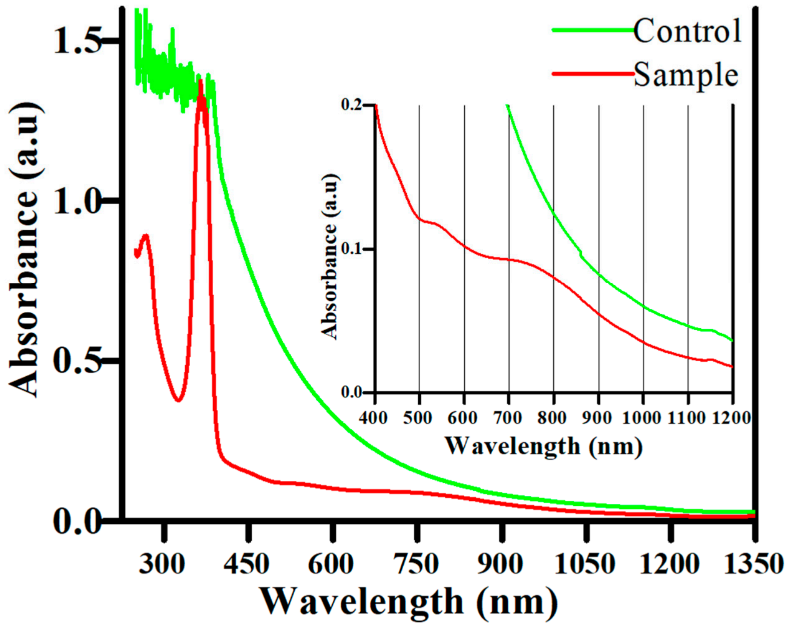

Figure S8: Absorption spectra of PNIPAm-*co*-allylamine microgel samples containing NIRAuNMs vs control microgels containing no AuNPs.
